# Caspase-2 regulates oncogene-induced senescence

**DOI:** 10.18632/oncotarget.2286

**Published:** 2014-07-31

**Authors:** Delphine Gitenay, Hélène Lallet-Daher, David Bernard

**Affiliations:** ^1^ Inserm U1052, Centre de Recherche en Cancérologie de Lyon, Lyon, France; ^2^ CNRS UMR5286, Lyon, France; ^3^ Centre Léon Bérard, Lyon, France; ^4^ Université de Lyon, Lyon, France

**Keywords:** caspase-2, senescence, cancer

## Abstract

Cellular senescence is activated by numerous cellular insults, in particular those driving cancer formation, resulting in stable proliferation arrest and acquisition of specific features. By self-opposing to oncogenic stimulation, senescence is considered as a failsafe program, allowing, when functional, to inhibit cancers occurrence. Compelling evidences suggest a tumor suppressive activity of caspase-2, eventually independently of its effect on cell death. The original results described here demonstrate that this tumor suppressive activity of caspase-2 is mediated, at least in part, by its pro-senescing activity. Indeed, we have demonstrated *in vitro* and i*n vivo* that loss of function of caspase-2 allows to escape oncogenic stress induced senescence. These results are discussed in the context of known tumor suppressive activity of caspase-2.

Caspase-2 is the most evolutionarily conserved of all the caspases. Nevertheless the caspase-2 knockout mice do not display major alteration and its role in apoptosis remains discussed [1]. Caspase-2 is regulating proliferation and transformation showing a role beyond apoptosis regulation. In particular, during these last years compelling evidence support an important tumor suppressive role of the caspase-2. Indeed, the loss of caspase-2 cooperates with Ras to transform mouse embryonic fibroblasts [2], with myc to favor lymphomagenesis [2, 3], or with neu to favor mammary tumorigenesis [4].

An important mechanism by which a tumor suppressor might exert its activity is by favoring senescence. During cell cycle arrest caused by tumor suppressors, mitogenic signaling like Ras and mTOR drive conversion of cell cycle arrest to senescence [5]. Indeed, to counteract an oncogenic signal and avoid the production of aberrant daughter cells, a normal cell can enter in a senescent state. This state results in a stable proliferation arrest and to the elimination of these dysfunctional cells by the immune system. This program is considered as a failsafe program that counteracts the oncogenic signal. Thus, the carcinogenesis process requires the loss of tumor suppressors to escape oncogene-induced senescence. The mechanisms involved in oncogene-induced senescence escape are still unclear, especially in epithelial cells [6].

In order to isolate loss of tumor suppressors favoring oncogene-induced senescence escape, we have recently performed a loss of function genetic screen [7]. To this end, we have used human mammary epithelial cells immortalized by expressing the hTert and expressing an inducible MEK oncogene (MEK:ER). These cells were infected by an shRNA library covering the complete genome and treated by 4-OHTamoxifen to activate MEK and induce senescence [7]. Short hairpins RNA against caspase-2 were identified in cells escaping senescence suggesting a role of caspase-2 in promoting oncogene-induced senescence failsafe program [7].

To validate the role of caspase-2, we generated two independent shRNA against the caspase-2 (Figure 1A) and we observed that they were both able to induce an escape from MEK-induced growth arrest (Figure 1B) and senescence as judged by the senescence associated-β-galactosidase activity (Figure 1C). In addition, the mature form of the caspase-2 was detected after the oncogenic stress and its appearance correlated with decreased cyclin A, a proliferation marker (Figure 1D). Importantly, caspase-2 knockout mice were more prone to develop skin tumor lesions in response to Ras activation (Figure 1E), these skin tumor lesions requiring escape from OIS as previously demonstrated in our laboratory [8]. An earlier study has already suggested a role of caspase-2 in regulating senescence as caspase-2 −/− mouse embryonic fibroblasts rapidly escape the senescence spontaneously occurring when these cells are cultivated in classical culture conditions [9].

**Figure 1:**
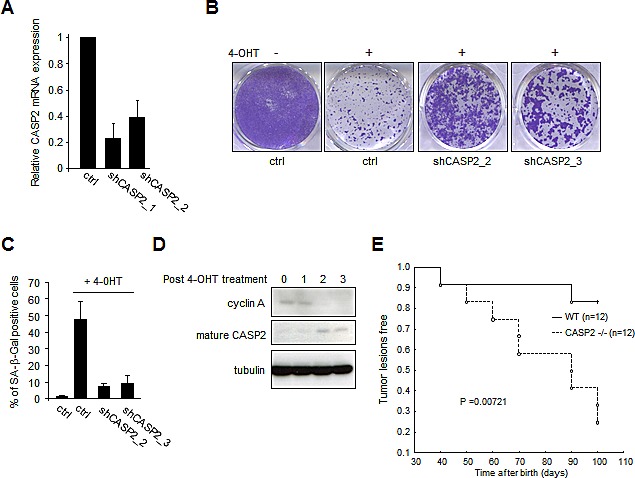
Caspase-2 loss allows oncogene-induced senescence escape (A-D) Normal human mammary epithelial cells were infected with retroviral vectors encoding the hTert (hygromycin resistance) and MEK:ER (neomycin resistance) and were selected. Cells were next infected by retroviral vectors encoding a control or an shRNA directed against the caspase-2 (puromycin selection) and selected. (A) RT-qPCR analysis of caspase-2 expression. The relative caspase-2 (CASP2) mRNA expression was normalized with respect to actin mRNA expression. (B) The same number of cells was seeded and treated daily three times with 4-OHT (400 nM) to activate MEK. Eight days after the first 4-OHT treatment, cells were PFA-fixed and cell growth was monitored with crystal violet-staining or, (C) they were assayed for their SA-β-Gal activity, a senescence marker. (D) Cells were treated with 4-OHT and cell extracts were prepared at the indicated times. They were next analyzed by immunoblotting with antibodies detecting the substrate of MEK, P-ERK, the cyclin A proliferation marker and the mature caspase-2. Tubulin levels were monitored to check protein loading. (E) Six weeks old mice were treated once by the carcinogen DMBA and then daily, twice a week, by the promoter TPA. Kaplan Meier analyses of the probability to develop tumor lesions were performed. P values were calculated using a log rank test.

Together these results demonstrate the ability of the caspase-2 to promote senescence. This strongly supports that the tumor suppressive activity of the caspase-2 is largely mediated by its ability to promote senescence.
